# Objectively Diagnosing Pulpitis: Opportunities and Methodological Challenges in the Development of Point-of-Care Assays

**DOI:** 10.3390/ijms27010355

**Published:** 2025-12-29

**Authors:** Darren Walsh, Ross Quigley, Anthonia Ekperuoh, Henry F. Duncan

**Affiliations:** Division of Restorative Dentistry & Periodontology, Dublin Dental University Hospital, Trinity College Dublin, Lincoln Place, D02 F859 Dublin, Ireland; darren.walsh@dental.tcd.ie (D.W.); ross.quigley@dental.tcd.ie (R.Q.); anthonia.ekperuoh@dental.tcd.ie (A.E.)

**Keywords:** pulpitis, next-generation diagnostics, caries, point-of-care assays, transcriptomics

## Abstract

Pulpitis is the inflammatory response of the dental pulp to microbial challenge and can range from mild to severe in nature, with severe pulpitis traditionally resulting in pulp removal and root canal treatment (RCT). In the pursuit of more conservative treatments, recent clinical practice guidelines have recommended strategies that preserve the vitality of the dental pulp, rather than RCT, when possible. This has increased the focus on improving the accuracy of pulp diagnosis, which will direct treatment and improve management outcomes. Unfortunately, current point-of-care (PoC) tools are subjective, lack discrimination and rely on the stimulation of pulpal neurons, limiting dentists’ ability to objectively identify the level of inflammation. Molecular biomarker assessment has the potential to dynamically analyse pulpitis and correlate this with inflammatory thresholds and treatment outcomes. Numerous chemokines, cytokines, proteases and growth factors exhibit altered expression during pulpitis and can be collected intraoperatively as part of routine dental treatment. Although current data indicate several markers that could be used as next-generation diagnostic chairside tools for pulpitis, there are currently no commercial kits. Considering the interest in vital pulp treatment, there is an urgent need to engage researchers, industry, dentists and other stakeholders in the development of PoC diagnostic assays for pulpitis.

## 1. Introduction

Dental caries and pulpitis remain pervasive and present significant clinical challenges. According to the World Health Organization, dental caries is one of the most prevalent non-communicable diseases globally, affecting individuals across all age groups [1]. Inflammation of the dental pulp (or pulpitis) often occurs as a result of deep caries and can be managed by a range of treatments, including vital pulp treatment (VPT), root canal treatment (RCT) or even extraction [2]. Recent endodontic guidelines and position statements from both the European Society of Endodontology (ESE) and the American Association of Endodontists (AAE) have advocated for more minimally invasive therapies that maintain the pulp tissue rather than removing it in an RCT procedure [2,3,4]. However, in order to conservatively manage deep caries and the exposed pulp and preserve pulp vitality, an accurate assessment of the condition of the pulp is essential [5].

**Figure 1:**
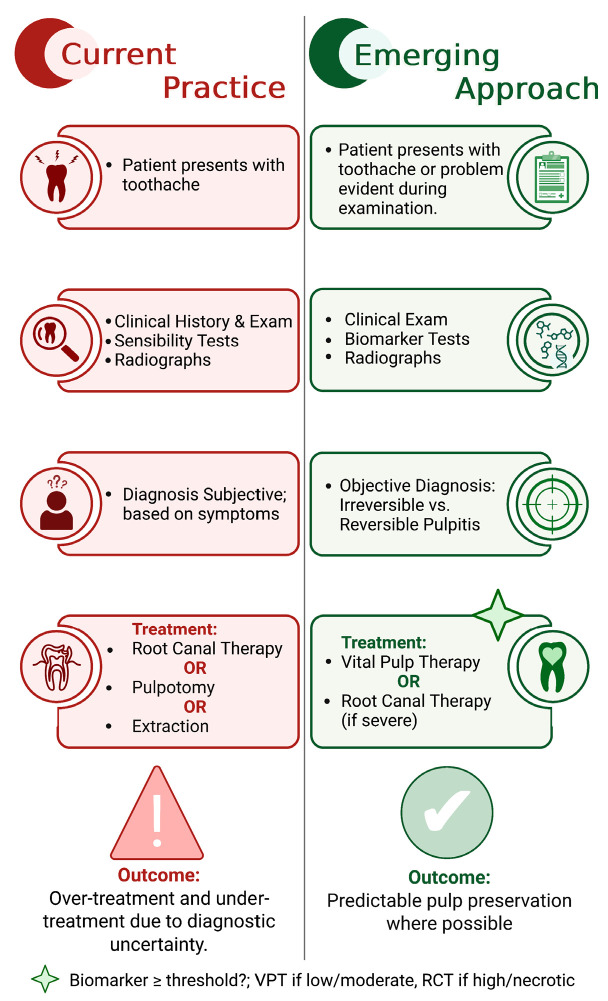
**Current versus emerging diagnostic–treatment workflows for pulpitis.** Conventional clinical practice relies on patient symptoms, sensibility testing (thermal/electric pulp tests) and radiographs to make diagnostic decisions. These approaches are subjective, leading to diagnostic uncertainty and potential overtreatment with root canal treatment (RCT) or extraction or undertreatment with vital pulp treatment (VPT). In contrast, an emerging biomarker-based approach integrates PoC molecular testing with routine clinical and radiographic assessment. Biomarker thresholds may objectively differentiate reversible from irreversible pulpitis, guiding treatment towards VPT, where inflammation is controlled, and reserving RCT for advanced pulpitis or necrotic disease. This approach aims to predictably preserve pulp vitality whenever possible and represents a paradigm shift in the management of pulpal disease.

Unfortunately, current diagnostic methods for the assessment of pulpitis are often crude and rely heavily on subjective measures such as patient history and clinical examination. This subjectivity can lead to variability in diagnosis and treatment outcomes, with under- and overtreatment evident [6] (Figure 1). Without accurate and objective diagnostic tools, the development and application of VPTs are hindered by a lack of predictability.

Traditional methods, including thermal and electric pulp tests, lack the specificity and sensitivity needed for an accurate, definitive diagnosis [7]. Recently proposed diagnostic solutions have advocated for the use of molecular biomarkers for the more objective diagnosis of pulpitis [8]. Biomarkers such as inflammatory cytokines, enzymes, matrix metalloproteinases and genetic material offer promising avenues towards enhancing the diagnostic accuracy [9]. Despite their potential, these molecular diagnostics have not been widely adopted in dental practice due to several practical limitations. Techniques like quantitative real-time PCR (qRT-PCR) and multiplex ELISA, while highly specific, require advanced laboratory equipment and technical expertise [10]. Furthermore, the analytes required for these tests are often only obtainable during invasive procedures, limiting their utility in routine preoperative diagnostics [11]. For these objective molecular diagnostics to have a translational impact, the development of economical, objective point-of-care (PoC) analytic tools is critical, as these represent dynamic diagnostic testing immediately at the time and location of dental care. These tools should ideally be capable of diagnosing pulpitis both preoperatively and intraoperatively, providing immediate and accurate real-time diagnostic information to inform treatment decisions (Figure 1). However, the current analytes and analytical tools employed in molecular diagnostics are not yet suitable for integration into PoC assays due to their complexity and cost, commercial inactivity and the lack of clinical validation of biomarkers [12].

This review aims to evaluate subjective and objective pulpal diagnostic tools from a translational perspective. It will consider the analytes and analytical techniques currently in use and explore the potential for developing practical PoC assays to improve the diagnosis and management of pulpitis.

## 2. Pulpitis

### 2.1. The Pulp in Health and Disease

Pulpitis is an inflammatory condition affecting the dental pulp, commonly triggered by bacterial invasion from caries or after dental trauma. The tooth is covered externally by enamel and is composed primarily of hydroxyapatite crystals formed by ameloblasts during tooth development [13]. Under the enamel is the dentine, which encases the pulp; this is a soft, highly vascularised tissue located at the tooth’s core. The pulp comprises several types of cells, including fibroblasts, odontoblasts and various immune cells [14]. Dentine is a mineralised tissue containing microscopic ‘dentinal’ tubules, which are produced by odontoblasts lining the interface between the dentine and the pulp [15] (Smith et al., 1995). Dentine, unlike enamel, is continually formed throughout life, with primary dentine formed during tooth development, secondary dentine formed throughout the life of the tooth and tertiary dentine in response to irritation [15].

Fibroblasts are the most abundant cells in the pulp, responsible for producing the extracellular matrix and collagen fibres, which constitute the majority of the pulp’s extracellular matrix (ECM) [16,17,18]. Odontoblasts form a continuous layer at the dentine–pulp border and are essential for dentin formation and repair [19]. The pulp also houses a rich supply of blood vessels and nerves, which enter through the apical foramen, providing nutrients and sensory function to the tooth [20]. The periodontal ligament interacts directly with the dental pulp at the apex and plays a critical role in supporting tooth structure and function [21]. The dentine and pulp are an interconnected tissue or complex, with dentine matrix proteins released from the ECM after injury or caries having been shown to support wound healing and dentine formation, indicating their potential in regenerative treatments for dental injuries [22]. Additionally, the pulp contains undifferentiated mesenchymal cells, capable of differentiating into odontoblasts or other cell types in response to injury [23] as part of a tertiary dentinogenesis process. The presence of immune cells, including macrophages and dendritic cells, highlights the pulp’s role in immune surveillance and response [24].

The onset of pulpitis begins when bacteria penetrate the enamel and infiltrate the dentine tubules, with their toxins diffusing down the dentinal tubules towards the pulp, triggering an immune response [25]. Pulp inflammation involves complex interactions between bacteria, immune cells and signalling molecules, leading to the characteristic signs and symptoms of pulpitis [26]. Bacterial toxins and metabolic byproducts stimulate the release of pro-inflammatory cytokines, such as interleukin-1β (IL-1β), tumour necrosis factor-alpha (TNF-α) and interleukin-6 (IL-6), as well as chemokines and other mediators from the resident pulp cells [14]. These cytokines activate local immune cells, including macrophages and dendritic cells, which further amplify the inflammatory response by recruiting additional immune cells such as neutrophils and T-lymphocytes to the site of infection [27]. Odontoblasts, lining the periphery of the pulp, also play a crucial sensory role by secreting antimicrobial peptides and signalling molecules that modulate the immune response [28].

As inflammation advances, increased vascular permeability leads to the exudation of plasma proteins and fluids into the pulp tissue, causing oedema and elevated intrapulpal pressure. This pressure can compress nerve fibres, resulting in the characteristic pain associated with pulpitis [29]. Research has shown that pulpitis can progress to necrosis without significant pain in many cases, significantly complicating the diagnosis and management of this condition [30]. If the infection is allowed to persist, the continued release of inflammatory mediators can lead to the degradation of extracellular matrix components by matrix metalloproteinases (MMPs) and the activation of apoptosis pathways in pulp cells, leading to tissue necrosis. Furthermore, the production of reactive oxygen species (ROS) by activated immune cells can cause oxidative stress, further damaging the pulp tissue. The extent and severity of pulpitis depends on the balance between the host’s defence mechanisms and the virulence of the invading pathogens. Without prompt intervention, pulpitis can progress to more advanced pulpitis, to localised necrosis and eventually to periapical abscess formation [5], underscoring the importance of early detection and treatment. Pulpitis can be halted and the pulp tissue preserved in a healthy condition if the stimulus is removed and appropriately restored [31]; this forms the basis of VPT strategies [6].

### 2.2. Prevalence and Impact of Pulpitis

Pulpitis is a common global dental condition primarily caused by bacteria in caries or microleakage, with its prevalence influenced by various demographic factors, including age, socioeconomic status and geographic location [32]. It has been highlighted that caries experience, a major precursor to pulpitis, increases with age and remains a significant issue in adults, despite declining rates in children [33]. This condition leads to significant economic impacts due to the high costs of dental treatments and the productivity losses associated with dental disease [34], with the estimated global economic burden of dental diseases, including pulpitis, being approximately USD 442 billion annually in 2010.

#### 2.2.1. Age and Socioeconomic Factors in Pulpitis

Age is a significant factor in the prevalence of pulpitis. As individuals age, the cumulative effects of dental caries and other oral health issues increase the risk of developing pulpitis. The DMFT (Decayed, Missing and Filled Teeth) index tends to rise with age, indicating a higher prevalence of dental caries, which can progress to pulpitis if untreated [33]. A study in a tertiary care hospital in South India found a high prevalence of pulpitis among adults, particularly among those aged 18–30 years, indicating that pulpitis is a significant concern in this demographic and that geographic location may be a modulating factor [35]. Research also highlights that the primary dentition is particularly affected by pulpitis, demonstrating the need for early prevention, education and continuous care [36].

Socioeconomic status also plays a crucial role in caries-induced pulpitis. A systematic review of European populations over time revealed significant variations in dental caries prevalence over time, influenced by what were considered socioeconomic factors [37]. Individuals from lower socioeconomic backgrounds often lack access to dental care, resulting in higher rates of untreated caries and pulpitis. These disparities highlight the need for targeted public health interventions to improve access to dental care and preventive measures in less advantaged populations. Studies have shown that social inequalities significantly impact oral health outcomes, with lower socioeconomic status linked to higher incidences of dental caries, pulpitis and endodontic sequelae [38]. This necessitates public health strategies aimed at reducing these disparities through increased access to preventive care and education. RCT is a time-consuming, expensive treatment that can be avoided by carrying out VPT in selected cases of caries exposure in the presence or absence of symptoms [2]. Although VPTs such as pulp capping and pulpotomy take less time and cost less than RCT [39], early VPT failure has been linked to misdiagnosis which highlights the potential benefit of PoC assays for the success of VPT. A challenge arises, however, in terms of the cost of these additional tests, as, although diagnostic tests may be relatively inexpensive, they are an additional expense that must be borne by the patient, insurer, health service or dentist, which may limit their uptake in lower socioeconomic groups.

#### 2.2.2. Geographic Variations in Pulpitis

In developing countries, higher rates of dental caries and pulpitis are driven by limited access to dental care, poor oral hygiene, the absence of dental education and a lack of fluoridated water. For example, there is a higher prevalence of dental caries in East Africa, with a notable impact on the population’s oral health [40]. Other studies have observed high rates of pulpitis and related dental diseases in regions with limited dental care infrastructure [41,42]. A global systematic review highlighted that early childhood caries remains a widespread issue, affecting almost half of preschool children worldwide, with significant variation between countries [43].

There have been attempts to address these geographic disparities by implementing community-based dental programs and increasing the availability of fluoride treatments. A study on the oral health of Irish adults revealed significant improvements in dental health outcomes due to increased access to preventive care and regular dental visits [44]. Additionally, policies aimed at improving access to dental services in rural and underserved areas are essential to reduce the burden of pulpitis and other dental diseases.

#### 2.2.3. Economic and Social Impacts of Pulpitis

The direct economic costs related to pulpitis include expenses related to dental treatments, such as dental restorations, RCT and extractions. Indirect costs arise from productivity losses due to dental pain and the time taken off work for dental visits. It has been estimated that dental diseases, including pulpitis, cost the global economy approximately USD 442 billion annually [34]. This figure underscores the significant financial burden that dental conditions impose on both individuals and healthcare systems.

Untreated caries and subsequent pulpitis can severely affect quality of life. The pain and discomfort associated with pulpitis can interfere with daily activities such as eating, speaking and sleeping and can significantly impact daily functioning [45]. Additionally, the potential for tooth loss due to untreated pulpitis and dental hard tissue destruction can lead to aesthetic concerns, impacting self-esteem and social interactions [42,46].

#### 2.2.4. General Health Implications of Pulpitis

The health implications of untreated pulpitis extend beyond the oral cavity. Downstream chronic dental infections including apical periodontitis can contribute to systemic conditions such as cardiovascular disease, diabetes and respiratory infections [47]. Therefore, the effective management of pulpitis is crucial not only in maintaining oral health but also for overall health and well-being.

## 3. Prevention and Management of Pulpitis

The profound societal and economic impacts of pulpitis necessitate effective prevention and management strategies. Global policies by the World Health Organization aim to integrate oral health into chronic disease prevention efforts, addressing the widespread impact of dental diseases [48]. Public health initiatives should prioritise increasing access to preventive care, promoting good oral hygiene practices and ensuring the timely treatment of dental caries to prevent progression to pulpitis. Regular dental check-ups, fluoride treatments and education on proper oral hygiene can significantly reduce the prevalence of pulpitis and its associated impacts [45].

### 3.1. Importance of Accurate and Timely Diagnosis

The accurate and timely diagnosis of pulpitis is crucial for effective treatment and predictable conservative management. Misdiagnosis or delayed diagnosis can result in the progression of the condition to more severe stages, necessitating more complex and costly treatments. Early detection allows for less invasive procedures and better outcomes, preserving the natural teeth and preventing further complications [49]. The importance of clinical and histologic correlations in diagnosing pulpitis accurately has been stressed [7]. Furthermore, a recent study highlighted that early and correct diagnosis can lead to cost-effective treatment options, such as pulpotomy, over more invasive procedures like RCT [50]. Moreover, advancements in objective molecular diagnostics are promising in improving the accuracy of pulpitis diagnosis, potentially reducing the reliance on subjective clinical assessments [51].

### 3.2. Current Treatment Modalities Based on Severity of Pulpitis

The management of pulpitis is based on the severity of the disease. For reversible pulpitis, non-invasive treatments such as indirect pulp capping or conservative management of the exposed pulp are typically employed. These VPT approaches involve removing carious tissue and applying a protective dressing to encourage healing and maintain pulp vitality [3,4]. VPTs for the management of the exposed pulp include pulp capping and partial and full pulpotomy procedures [3]. Pulp capping techniques have evolved significantly in recent years, with modern materials such as hydraulic calcium silicate cements (HCSCs) demonstrating high success rates if the teeth are well restored afterwards [52]. Long-term studies show that direct pulp capping can be effective, with a survival rate of 76.3% over 13.3 years, suggesting that it is a viable option in managing pulpitis [53]; however, this appears to be operator-dependent as other, more pragmatic studies have shown significantly lower results of under 50% after 5 years for pulp that is caries-exposed [54]. In contrast, teeth displaying signs and symptoms of irreversible pulpitis, where the pulp is severely inflamed and cannot heal, are recommended to undergo more invasive procedures such as pulpotomy or pulpectomy [7]. Pulpotomy, which involves the removal of pulp tissue—i.e., 2–3 mm in the case of partial pulpotomy and the entire coronal pulp in the case of a full pulpotomy—and pulpectomy are possible treatments for irreversible pulpitis [46]. Although pulpotomy procedures have recently demonstrated high success in the management of teeth with a clinical diagnosis of irreversible pulpitis [55], the failure of such procedures is generally linked to the incorrect assessment of the pulpitis severity preoperatively [56]. Therefore, developing new objective diagnostic tools that can accurately assess the state of the pulp are critical if we are to improve the predictability and reliability of VPT procedures.

## 4. Diagnostic Tools for Pulpitis

There is a range of tools used to determine the presence or absence of pulpal disease; many of these are subjective tests used to elicit pain from the tooth, while others include imaging or histological analysis.

### 4.1. Traditional Diagnostics for Pulp Disease

#### 4.1.1. Clinical Symptoms and Pain Assessment

Pulpitis diagnosis often begins with an assessment of clinical symptoms and history. Classically, reversible pulpitis presents with mild to moderate pain that is provoked by stimuli and subsides quickly, while, in contrast, irreversible pulpitis often involves severe, lingering pain that can occur spontaneously [57]. However, relying solely on symptoms can be subjective, as the pulp may become necrotic asymptomatically [30] and, if symptoms are present, they may not accurately reflect the pulp’s condition [58]. Furthermore, assessment of the tooth may involve tenderness to percussion; however, the response to these tests is dynamic and crude and has been shown not to be indicative of the response to treatment [59].

#### 4.1.2. Pulp Sensibility Testing

Current pulp tests are principally used to assess pulp vitality, i.e., whether the dental pulp is vital or necrotic, and they achieve this by using thermal stimuli or electric currents to assess the neurogenic response in the pulp [3]. Diagnostic tests that measure true vitality, i.e., whether a blood supply remains in the tooth, such as laser Doppler or pulse oximetry, have shown promise but, at present, are not available commercially [2]. An exaggerated response to heat, cold or electricity may be used to indicate the level of pulpitis, but this is crude and the results are subjective [49]. Chairside testing using thermal or electric pulp tests is also limited when teeth are heavily restored or the pulp tissue has excessive tertiary dentine formation; this may result in false negatives and potential overtreatment. Conversely, necrotic pulp may remain responsive in certain patients due to connections through neighbouring teeth or gingival tissue or even in anxious patients, exhibiting a false positive response.

#### 4.1.3. Imaging Techniques

Radiographs (bitewing and periapical) are commonly used as part of the diagnosis of pulpitis as they can give an indication of the bacterial depth in the tooth and indicate whether a periapical radiolucency is present. Recently, there have been efforts to categorise the depth of caries, presenting the bacterial front and, concomitantly, the level of pulpal inflammation from the radiographic image, classifying the lesion as ‘deep’ or ‘extremely deep’ [3], with extremely deep lesions associated with more severe pulp inflammation and even infection [60]. However, radiographic imaging has its limitations, being influenced by film processing and beam angulation, as well as only showing hard tissue changes and not inflammatory changes within the pulp [61]. Cone-beam computed tomography is not recommended for the assessment of the pulpal inflammatory state [3], but dental MRI may be useful in the future in this regard [62]. Furthermore, bitewing and periapical radiographs provide 2D representations of 3D anatomical structures, resulting in geometric distortion and anatomical noise [63]. Endodontic imaging techniques contribute indirectly to the diagnosis of pulpitis through the identification of associated structural changes such as periapical radiolucency, loss of the lamina dura and widening of the periodontal ligament space [64]. Moreover, the interpretation of both conventional radiographs and CBCT images is influenced by intra- and interoperator and -examiner variability. The incorporation of a direct, objective measure of pulpal inflammation would therefore represent a significant advancement in the endodontic diagnostic workflow.

#### 4.1.4. Histological Analysis

Histological examination remains the reference standard in confirming the presence and location of pulpal inflammation, providing detailed insights into the inflammatory status of the pulp tissue. However, it only represents a ‘snapshot’; it is invasive and not feasible for routine clinical practice due to the need for tooth extraction, highlighting the need for more practical diagnostic tools [7]. A core problem in the assessment of pulpitis is the absence of a reliable clinical referenced standard [49]. This presents an opportunity for the development of an objective reference standard using a molecular signature of pulpitis that could be analysed in a PoC test. By shifting pulpitis assessment from the subjective interpretation of symptoms and indirect imaging findings towards quantifiable biological markers, future diagnostic strategies may allow for improved objectivity, consistency and diagnostic accuracy in endodontic practice.

### 4.2. Molecular Diagnostics for Pulpitis

The biochemical alterations that are evident in the pulp and surrounding tissues offers an opportunity for the collection of a tissue fluid such as pulpal blood, dentinal fluid or gingival crevicular fluid, which could be objectively measured to highlight the inflammatory level in the pulp [9] (Table 1). As described in the following section, a plethora of markers have been used in clinical studies (Table 1). The interpretation of pulpal biomarkers across the currently available literature is complicated by methodological heterogeneity. Differences in the sample source (e.g., coronal pulp tissue, pulpal blood, dentinal fluid or periapical exudates), analytical approach (gene expression versus protein quantification) and biological context (in vitro, ex vivo or clinical studies) may contribute to variability in the reported biomarker profiles. In addition, patient- and tooth-related factors such as age, dentition type, caries extent and treatment indication are often addressed through study design and inclusion criteria rather than formal statistical adjustment. These considerations should be taken into account when comparing biomarker performance across studies and highlight the critical need for standardised methodologies in future validation work.

#### 4.2.1. Molecular Origins of Biomarkers for Pulpitis

**Figure 2:**
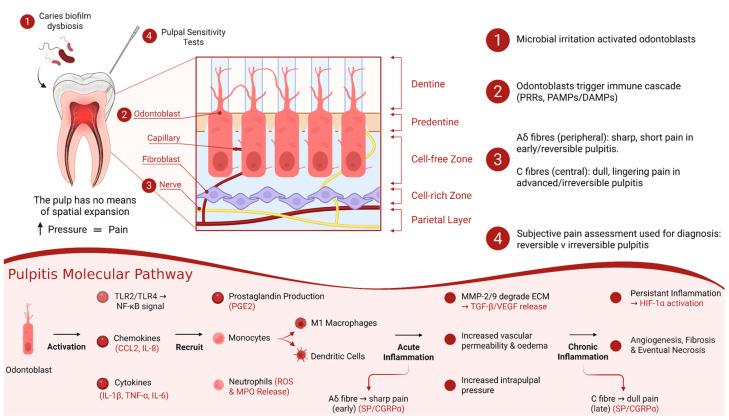
**Molecular pathogenesis of pulpitis.** Microbial metabolites from caries-associated dysbiosis diffuse through dentinal tubules and activate odontoblasts via TLRs, triggering NF-κB signalling and the release of cytokines (IL-1β, TNF-α, IL-6) and chemokines (CCL2, IL-8). These mediators recruit neutrophils, macrophages and dendritic cells, driving acute inflammation marked by ROS production, MMP activation and prostaglandin synthesis. Persistent inflammatory activity promotes vascular permeability, hypoxia and the release of HIF-1α-driven VEGF, contributing to angiogenesis and fibrosis. Neurogenic inflammation arises through the release of substance P and CGRPα from C-fibres, while Aδ-fibres transmit early sharp pain responses. The combined molecular and neurogenic signalling shapes the measurable biomarker profile, highlighted with red text in the above figure, of pulpitis, providing an objective framework for diagnosis beyond subjective clinical assessment.

The intricate interplay among various resident pulp cells, including odontoblasts, fibroblasts, dental pulp stem cells and immune cells, contributes to significant alterations in the biomarker profile during inflammation, which supports robust diagnostic methods for pulpitis [86,87]. Of these cells, odontoblasts constitute the first line of defence against bacteria present in caries, long before the bacteria have reached the pulp chamber, whereby metabolic byproducts and bacterial toxins can diffuse through the dentinal tubules and interact with odontoblasts at the pulp–dentine interface [88]. Odontoblasts express pattern recognition receptors (PRRs) capable of detecting pathogen- and damage-associated molecular patterns (PAMPs and DAMPs) [89]. Among PRRs, the Toll-like receptors (TLRs), particularly TLR2 and TLR4, initiate the innate immune response by sensing conserved molecular patterns for the early immune recognition of pathogens in pulpitis [65,90]. Upon the detection of bacterial components such as lipoteichoic acid or lipopolysaccharide (LPS), TLR activation triggers the translocation of nuclear factor κB (NF-κB), further potentiating PRR activation and the release of downstream chemokines, including CCL2 and IL-8 [65,89] (Figure 2).

CCL2, also known as monocyte chemoattractant protein 1 (MCP-1), is induced by inflammatory stimuli and promotes the extravasation of effector cells from the local vasculature across the endothelium [70]. These migrating monocytes differentiate into pro-inflammatory M1 macrophages and dendritic cells (DCs), contributing to antigen presentation and cytokine amplification. M1 macrophages secrete IL-1β, TNF-α, IL-6 and nitric oxide, enhancing microbial clearance but also sustaining inflammation [91]. In parallel, DCs play a specialised role in linking innate immune function with the induction of adaptive immunity by increasing the expression of major histocompatibility complex (MHC) class II molecules and loading antigenic peptides onto these MHC class II molecules before presenting them to naïve T cells [92,93]. TNF-α, which is markedly elevated in irreversible pulpitis [94], amplifies vasodilation, tissue injury and vascular permeability via direct and indirect bradykinin-mediated effects [67]. Additionally, IL-6 and IL-1β drive prostaglandin synthesis through the NF-κB- and MAPK-mediated transcription of cyclooxygenase-2 (COX-2), while concurrently stimulating odontoblast activity and leukocyte infiltration [68,69,95]. Prostaglandins, especially PGE2, are formed via the COX-mediated metabolism of arachidonic acid and reinforce inflammation through a positive feedback loop with IL-6 [66].

Neutrophils, abundantly recruited to the dental pulp during pulpitis, represent key early responders in innate immune defence [96]. Neutrophil infiltration is driven by chemokines and pro-inflammatory mediators, such as IL-6, released following PRR activation on odontoblasts and macrophages [97,98]. Once recruited, neutrophils contribute to microbial clearance through phagocytosis, the secretion of ROS and proteolytic enzymes such as pulpitic marker MMP-9 [53] and the release of extracellular traps embedded with antimicrobial peptides [99]. Additionally, increased ROS levels activate p38 MAPK signalling, facilitating myeloperoxidase (MPO) release via neutrophil degranulation, representing a promising biomarker indicative of inflammation and oxidative stress [100].

Matrix metalloproteinases (MMPs) are proteolytic enzymes that are integral to extracellular matrix (ECM) remodelling and immune signalling [101]. MMP-8, also known as neutrophil collagenase, is predominantly released by activated neutrophils and contributes to collagen degradation during inflammatory ECM remodelling. Collagen represents a major structural component of the dental pulp, and increased MMP-8 expression has been demonstrated in inflamed pulpal and periapical tissues, as well as in root canal exudates, with levels shown to decrease following successful endodontic treatment [72]. Clinical studies have reported significantly elevated MMP-8 concentrations in teeth diagnosed with irreversible pulpitis compared with healthy pulp, with no consistent difference observed between symptomatic and asymptomatic cases, suggesting that MMP-8 reflects inflammatory activity rather than pain perception [73]. In addition, MMP-8 levels have been shown to correlate with prolonged responses to cold sensibility testing, supporting its potential role as an adjunctive biomarker in the objective assessment of pulp inflammation [102]. Neutrophils, macrophages and fibroblasts secrete inactive pro-MMP-9, which becomes activated through proteolytic cleavage [74]. Activated MMP-9 contributes to ECM degradation, chemokine activation and the modulation of vascular permeability [103]. MMP-2, predominantly associated with neutrophil activity, facilitates necrosis and monocyte diapedesis by transiently regulating endothelial junctions [75]. Collectively, MMP-2 and MMP-9 promote cellular infiltration by releasing latent growth factors, such as TGF-β and vascular endothelial growth factor (VEGF), thereby further propagating inflammation and angiogenesis and presenting translational opportunities as measurable biomarkers in the objective diagnosis of pulpitis [53,55].

Persistent inflammatory microenvironments induce interstitial hypertension, haemodynamic disturbances, ischaemia and hypoxia [104]. Under these conditions, the activation of hypoxia-inducible factor-1α (HIF-1α) occurs across several pulpal cell types [105]. In most tissues, HIF-1α activation promotes compensatory vasodilation and enhanced oxygen delivery; however, the rigid dentinal encasement of the pulp prevents volumetric expansion, thereby exacerbating oedema and creating intrapulpal pressure [76]. Controlled HIF-1α activity upregulates VEGF transcription, driving neovascularisation and facilitating leukocyte trafficking. This response can be protective in reversible pulpitis but becomes maladaptive as inflammation persists, contributing to chronic hypoxia, fibrosis and eventual necrosis [76]. Additionally, hypoxic and inflammatory conditions within the rigid pulpal environment also promote metabolic reprogramming, with an increased reliance on anaerobic glycolysis and subsequent lactate accumulation [82]. Concurrently, excessive ROS production disrupts redox haemostasis, leading to the adaptive upregulation of antioxidants such as glutathione [81]. Alterations in lactate and glutathione thereby reflect metabolic and oxidative stress on the inflamed pulp and represent complementary biomarkers of inflammatory activity and the tissue response.

Local complement activation represents a key molecular link between inflammation and repair within the dental pulp. Contrary to the traditional view of complements as a liver-derived plasma cascade, recent work has shown that pulp fibroblasts themselves can synthesise and secrete functional complement components, including C3a, C5a and the membrane attack complex (MAC) [77]. Beyond mediating microbial clearance through opsonisation and lysis, these complement-derived fragments promote pulp regeneration [78]. C3a stimulates fibroblast and stem cell proliferation, while C5a enhances DPSC recruitment, odontoblastic differentiation and neurite outgrowth [106,107,108,109]. The presence of complement fragments in pulpal and periapical fluids exhibits translational potential as a biomarker that reflects both inflammatory activity and the regenerative response.

MicroRNAs (miRNAs) represent an additional regulatory layer in pulpitis, acting as short non-coding RNAs that bind target mRNAs to repress translation and promote mRNA degradation [110,111]. Emerging evidence suggests that altered miRNA expression contributes to the balance between inflammatory activation and reparative responses in the dentine–pulp complex, including the modulation of immune signalling and dental pulp cell differentiation [83,84]. While miRNAs show promise as diagnostic and prognostic biomarker candidates in pulpitis, their clinical utility may be limited by issues of specificity and network-wide regulatory effects, and standalone miRNA signatures may require integration with complementary biomarkers to enhance the diagnostic accuracy [85].

Within the dental pulp, nociceptive afferents comprise both thinly myelinated Aδ-fibres and unmyelinated C-fibres. Aδ-fibres conduct fast, sharp, well-localised pain, characteristic of early or reversible pulpitis. In contrast, C-fibres conduct slower, dull, lingering pain, which is typically associated with advanced or irreversible pulpitis [29]. Many of these nociceptors contain neuropeptides, such as substance P (SP) and calcitonin gene-related peptide (CGRPα), which contribute to vasodilation, plasma extravasation and the activation of immune cells [80]. Increased levels of SP and CGRP in pulpal and periapical fluids have been associated with the severity of inflammation and pain intensity. However, these mediators reflect general neurogenic inflammation rather than specific fibre activation [79]. Consequently, while neuropeptide concentrations may serve as supportive biomarkers of disease activity, they are not reliable indicators of the stage of pulpitis due to their lack of specificity.

Collectively, these molecular events underpin the biomarker profiles measurable in pulpal and periapical fluids. Their differential expression across disease stages forms the mechanistic basis for emerging molecular biomarkers in the robust diagnosis of pulpitis (Figure 2). Table 1 summarises the significant biomarkers identified within these pathways, their cellular sources and their relevance to clinical diagnosis.

#### 4.2.2. Types of Biomarkers

**Figure 3:**
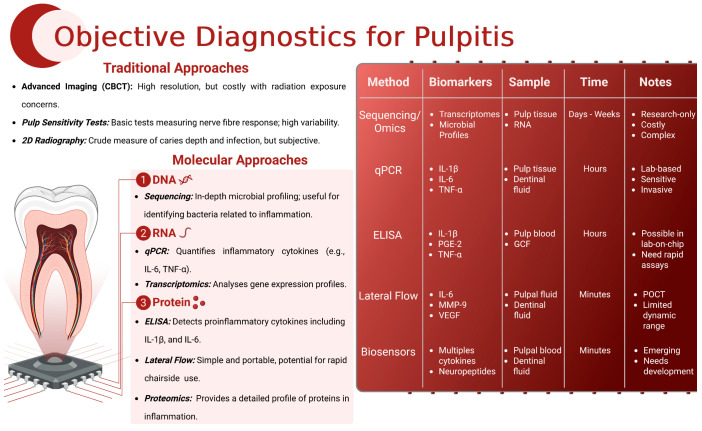
**Comparative overview of traditional and molecular diagnostic approaches for pulpitis.** Traditional pulp diagnostic methods, including sensibility testing and 2D radiography, and, more recently, cone-beam computed tomography (CBCT), offer only indirect or late-stage insights into pulpal status. Molecular approaches target the underlying biology at the level of nucleic acids and proteins, enabling the objective and quantitative diagnosis of pulpitis. Sequencing and transcriptomics provide detailed microbial and host profiles but remain costly and research-focused. qPCR enables the sensitive detection of cytokine transcripts (IL-1β, IL-6, TNF-α) but requires invasive sampling and laboratory infrastructure, which is unfeasible for current dental practice. ELISA offers quantitative protein analysis (e.g., IL-1β, PGE_2_, TNF-α) but is currently constrained by assay turnaround. Translational platforms such as lateral flow assays and biosensors (microfluidic and electrochemical) can detect inflammatory mediators (e.g., IL-6, MMP-9, VEGF, neuropeptides) in pulpal fluid or dentinal samples within minutes, offering the greatest promise for the objective, chairside diagnosis of pulpitis.

The sampling of biomarkers, including DNA, RNA and proteins, has shown promise in enhancing the accuracy and objectivity of pulpal diagnosis. These molecular indicators can provide insights into the biological processes underlying pulp inflammation, potentially allowing for earlier and more precise diagnosis [9] (Figure 3).

##### DNA-Based Biomarkers

Genetic markers associated with pulpitis, detected using PCR and qPCR, offer high specificity. These techniques can detect specific pulpitis-related genetic sequences but require specialised equipment and expertise, limiting routine clinical use, particularly at chairside, where results are required in a short timeframe [10].

##### RNA-Based Biomarkers

Candidate RNA-based biomarkers, identified through transcriptomic profiling, provide valuable information about gene expression patterns associated with pulpitis. Techniques such as qPCR can reliably quantify these biomarkers, offering insights into the disease’s progression and helping to differentiate between reversible and irreversible pulpitis [50]. However, as was evidenced in the COVID-19 pandemic, although reliable, RNA-based techniques require processing times of several hours, again limiting their clinical dental PoC applicability (Figure 3).

##### Protein-Based Biomarkers

Proteins involved in the inflammatory response, such as cytokines and enzymes, have been studied extensively—either a candidate ELISA approach [55], profiling antibody array [112] or proteomic investigation [8]. Key proteins such as IL-1β, TNF-α and osteocalcin are significantly elevated in pulpitis and can be detected using methods like Western blot and ELISA. These protein biomarkers are particularly promising, not only for non-invasive diagnostics but also as rapid intraprocedure tests including analytes such as dentinal fluid and pulpal blood [11,55,113].

#### 4.2.3. Methods of Detection

##### Polymerase Chain Reaction (PCR) and Quantitative PCR (qPCR)

PCR and qPCR are highly sensitive and cost-effective techniques to amplify and quantify DNA and RNA sequences. These methods provide high sensitivity and specificity, making them ideal in detecting genetic and transcriptomic biomarkers associated with pulpitis. However, their requirements in terms of sampling, transport, specialised equipment and technical expertise limit their widespread use in routine dental practice, both preoperatively and intraoperatively [9].

##### Western Blotting

Western blotting is a common laboratory technique used to detect specific proteins within a sample. This technique separates proteins by electrophoresis, transfers them to a membrane and detects them using antibodies. This method is highly specific and can confirm the presence of protein biomarkers associated with pulpitis, although it is time-consuming and requires extensive sample preparation; for these reasons, it is generally confined to research projects and discovery research rather than clinical diagnosis [11].

##### Enzyme-Linked Immunosorbent Assay (ELISA)

ELISA is widely used to quantify one or several protein levels in a sample. It employs antibodies to detect and measure specific proteins, including pro-inflammatory cytokines or chemokines involved in pulpitis. ELISA is suitable for large-scale screening and provides high sensitivity, making it a valuable tool for diagnosing pulpitis, although, usually, obtaining quantifiable results takes at least 1 h using this technique, which limits its PoC utility in dental practice [114].

##### Lateral Flow Tests

Lateral flow tests work in a similar manner to ELISAs and were popularised during the COVID-19 pandemic [115]. The patient sample is applied in a buffer to a cellulose or other strip and allowed to diffuse along it until it reaches the position where the antigen binds and is immobilised. Antibodies specific to the antigen of interest bind and are visualised using a labelled antibody that facilitates rapid detection. The lateral flow test is attractive as a PoC tool as it is quick, easy and relatively cheap; however, it is not quantitative and is subject to false negatives and positives. Moreover, it requires commercial and industrial support to develop the kit, which, to date, has not occurred in operative dentistry for the chairside diagnosis of pulpitis [116].

##### Transcriptomics

Transcriptomic approaches involve high-throughput sequencing to analyse gene expression profiles. This method can identify novel biomarkers and provides detailed insights into the molecular mechanisms underlying pulpitis (see Section 4.2.1). The profiling nature of transcriptomics has the potential to transform biomarker discovery and enhance the diagnostic accuracy [50].

##### Proteomics

Proteomics encompasses a wide range of techniques designed for the large-scale analysis of proteins. Proteomic techniques include, but are not restricted to, protein arrays and mass spectrometry, and these high-throughput assays are used to study the proteome (i.e., all proteins expressed by a cell or tissue) [8]. Although these techniques offer new insights into protein expression during pulpitis, they should be used principally in the profiling or discovery of pulpitic biomarkers so as to guide future targeted approaches, as the use of sophisticated analytical techniques precludes their use at the chairside [117].

### 4.3. Point-of-Care Diagnostics for Pulpitis

#### 4.3.1. Current State of Point-of-Care (PoC) Diagnostics in Dentistry

PoC diagnostics in dentistry are limited to basic tests, such as thermal and electric pulp sensibility tests, which lack specificity, are subjective and cannot be used to determine the threshold of pulpitis or provide detailed diagnostic information [118]. There is a need for clinicians to know the threshold for reversible tissue damage so that tissue that is beyond salvage can be removed and the deeper healthier tissue stimulated to heal. At present, there are no commercially available objective PoC diagnostics for the determination of pulp disease. However, with advances in the provision of VPT [6], there is a significant need for more advanced, rapid and accurate diagnostic tools [49].

#### 4.3.2. Potential of Biomarker-Based PoC Diagnostics

Biomarker-based diagnostics offer the potential for rapid, non-invasive and accurate chairside testing. These tools could transform dental practice by providing immediate diagnostic results, improving treatment decisions and enhancing patient outcomes. Examples from other fields, such as lateral flow assays and biosensors, illustrate the feasibility of these technologies [50]. Table 1 highlights the range of potential inflammatory and other biomarkers that could be used in a single or combination PoC assay.

#### 4.3.3. AI-Assisted Biomarker Integration in PoC Diagnostics

Current PoC diagnostic frameworks are guided by the updated REASSURED guidelines, prioritising real-time connectivity, ease of specimen collection, affordability, sensitivity, specificity, user-friendliness, rapid and robust results, equipment-free modalities and deliverability to end-users. However, PoC-based platforms are limited by reduced diagnostic accuracy, sensitivity and precision when compared with conventional laboratory-based tools, such as qRT-PCR and multiplex biomarker detection [119].

Artificial intelligence (AI) offers a potential strategy to address some of these limitations by enabling the integration and interpretation of complex, multiplex biomarker data within PoC workflows. Machine learning (ML) approaches are designed to identify non-linear relationships across high-dimensional datasets, such as those commonly seen in healthcare, and, therefore, can support the combined analysis of multiple biomarkers, rather than relying on the thresholds of single, candidate analytes. Current healthcare-driven AI applications often focus on single modalities, unlike practitioners, who rely on the combination of multiple sources for informed clinical decision-making. Thus, a shift towards multimodality in AI-powered diagnostics is potentiating a significant transformation in PoC frameworks. For instance, AI-powered biosensors have been developed for the continuous monitoring of physiological parameters such as movement, heart rate, blood glucose and sleep patterns [120]. In the context of AI-powered PoC diagnostic assays for pulpitis, increased pattern recognition in large biomarker panels, alongside current testing modalities, such as imaging data, may revolutionise endodontics’ diagnostic accuracy. Importantly, interindividual genetic background and baseline inflammatory status are likely to influence biomarker expression; therefore, integrative AI-driven analyses of multiplex biomarker panels may help to mitigate biological variability and improve the diagnostic robustness across heterogenous patient populations.

Another method by which AI and ML can be integrated into the design of these PoC platforms is in AI-powered biomarker discovery. With the rapid increase in the availability of large multi-omics and multi-modal healthcare datasets, AI algorithms have become a cornerstone in the discovery of new biomarkers [121]. This means not only the discovery of diagnostic biomarkers but also risk, monitoring, prognostic, predictive and responsive biomarkers from samples that can be obtained during routine dental visits and tracked over time [122]. However, while this presents unique opportunities for large strides in healthcare approaches, limitations such as data handling, privacy, storage and over-reliance on AI to interpret and analyse multiple forms of clinical data arise.

#### 4.3.4. Challenges and Future Directions for the Development of PoC Diagnostics

The introduction of biomarker-based PoC diagnostic assays into endodontic practice may offer distinct advantages in a range of clinical and educational settings. In emergency clinics, the availability of a rapid, quantitative chairside test could support more informed clinical decision-making, minimally invasive treatment solutions and timely management. In general dental practice, such approaches may deliver personalised approaches by enhancing diagnostic consistency and reducing intra- and interpractitioner variability, thereby limiting the reliance on symptom-dependent diagnoses and reducing the use of unnecessary or overly invasive treatments. Within dental education, the use of objective diagnostic tools can enhance students’ and clinical staff’s understanding of the biological basis of dental pulpitis, while offering objective supports to implement clinical recommendations [2].

The development of PoC diagnostic tools for pulpitis faces several challenges, including technical and logistical barriers, cost constraints and the need for user-friendly devices. PoC implementation would require an initial financial investment, both in device acquisition and in training costs for students and practitioners. Furthermore, there is a risk of developing an over-reliance on such tools; therefore, biomarker-based PoC diagnostics should be considered adjuncts to existing diagnostic methods, rather than replacements, for comprehensive clinical examination alongside professional judgement. Further research and development are required to validate biomarkers and create reliable, portable diagnostic devices. Regulatory approval, industrial buy-in, clinical validation and the resolution of scalability issues are essential in bringing these innovations to market [20]. Other issues in developing PoC assays include the volumes of analytes available to dentists, where dentinal fluid and pulpal blood (µL) do not yield large quantities of substrate in comparison with systemic blood (mL). It is notable that systemic blood is generally not a useful substrate for pulpitis as most of the markers, e.g., IL-6, are produced locally in the pulp tissue and not systemically in the liver, e.g., CRP, and therefore will not be identified as upregulated in systemic blood [6].

## 5. Conclusions

The objective chairside detection of molecular biomarkers holds significant potential in improving the diagnosis of pulpitis, enabling earlier and more accurate detection compared with traditional methods. Integrating molecular diagnostics into clinical practice can enhance the precision of treatment decisions and outcomes [9].

Affordable PoC diagnostics could transform pulpitis care by making advanced diagnostic technologies accessible to more patients and clinicians in general dental practice. These tools would allow rapid, accurate diagnoses, leading to improved dental care, patient outcomes and ultimately oral health [12].

Further research, however, is required to discover and validate new biomarkers and develop practical diagnostic tools. Collaboration between researchers, clinicians and industry will be crucial in advancing these technologies and improving dental care. The future of pulpitis diagnosis linked to VPT looks promising, with the potential for significant advancements that could transform patient care and outcomes [6].

## Figures and Tables

**Table 1:** **Key molecular biomarkers in pulpitis and their potential diagnostic relevance.** This table summarises the principal molecular mediators involved in the inflammatory and regenerative processes of pulpitis. Each biomarker is listed with its predominant cellular source, core functional role and clinical relevance across disease stages. Together with Figure 1, the table highlights how these measurable molecular profiles provide a more objective diagnostic framework than current subjective clinical assessments.

Biomarker	Source	Function	Stage	Diagnostic Relevance	Reference
**IL-1β**	Odontoblasts Macrophages	Amplify inflammation Prostaglandin synthesis Endothelial permeability	Rev → Irrev	Reflects inflammatory burden	[65,66]
**TNF-α**	Macrophages DCs	Induce vasodilation Vascular leakage Tissue injury	Irrev	Marker of severe inflammation	[67]
**IL-6**	Odontoblasts Fibroblasts	Upregulate COX-2 Recruit neutrophils	Rev → Irrev	Sensitive early-stage marker	[68,69]
**CCL2**	Odontoblasts Fibroblasts	Recruit monocytes/macrophages	Early	Monocyte activation marker	[70]
**IL-8**	Odontoblasts Fibroblasts	Neutrophil chemotaxis	Early	Acute inflammation indicator	[71]
**MMP-8**	Neutrophils	Collagen degradation ECM remodelling	Rev → Irrev	Reflect collagen breakdown and inflammatory progression	[72,73]
**MMP-9**	Neutrophils Macrophages	ECM degradation Cytokine activation	Irrev	Tissue destruction marker	[74]
**MMP-2**	Neutrophils Fibroblasts	Endothelial junction regulation Necrosis	Irrev	Vascular injury marker	[75]
**MPO**	Neutrophils	ROS-linked degranulation Oxidative stress	Early	Inflammation and stress marker	[76]
**VEGF**	Fibroblasts Endothelium	Angiogenesis Vascular permeability	Late	Hypoxia and repair marker	[76]
**HIF-1α**	Odontoblasts Fibroblasts	Regulate VEGF under hypoxia	Late	Chronic hypoxia indicator	[76]
**C3a/C5a**	Fibroblasts	Recruit DPSCs Microbial clearance	Rev/Regen	Dual inflammation–repair marker	[77,78]
**MAC**	Fibroblasts	Microbial clearance	Rev	Microbial control	[77,78]
**SP**	Aδ/C-Fibres	Nociception Vasodilation	Irrev	Correlate with pain intensity	[79]
**CGRPα**	Aδ/C-Fibres	Nociception Plasma extravasation Vasodilation	Irrev	Correlate with pain intensity	[80]
**GSH**	Fibroblasts	Antioxidant defence ROS buffering	Rev → Irrev	Reflect oxidative stress balance and inflammatory burden	[81]
**Lactate**	Inflamed Pulp Cells	Markers of anaerobic metabolism Reflect hypoxia	Irrev	Indicator of metabolic stress and tissue hypoxia	[82]
**miRNA**	Pulp Cells Immune Cells	Post-transcriptional regulation Inflammatory modulation	Rev → Irrev	Epigenetic indicators of inflammation stage and progression	[83,84,85]

## Data Availability

No new data were created or analysed in this study. Data sharing is not applicable to this article.

## Abbreviations

AI	Artificial intelligence
ML	Machine learning
miRNA	MicroRNA
PRRs	Pattern recognition receptors
PAMPs	Pathogen-associated molecular patterns
DAMPs	Damage-associated molecular patterns
TLRs	Toll-like receptors
NF-kB	Nuclear factor kB
MCP-1	Monocyte chemoattractant protein 1
DCs	Dendritic cells
MHC	Major histocompatibility complex
COX2	Cycloxygenase-2
MPO	Myeloperoxidase
MMP	Matrix metalloproteinase
ECM	Extracellular matrix
VEGF	Vascular endothelial growth factor
HIF-1α	Hypoxia-inducible factor-1α
MAC	Membrane attack complex
CGRPα	Calcitonin gene-related peptide
SP	Substance P
